# Innate immune responses through Toll-like receptor 3 require human-antigen-R-mediated *Atp6v0d2* mRNA stabilization

**DOI:** 10.1038/s41598-019-56914-w

**Published:** 2019-12-31

**Authors:** Mohd Izwan Bin Zainol, Takumi Kawasaki, Warunthorn Monwan, Motoya Murase, Takuya Sueyoshi, Taro Kawai

**Affiliations:** Laboratory of Molecular Immunobiology, Division of Biological Science, Graduate School of Science and Technology, Nara Institute of Science and Technology (NAIST), Nara, 630-0192 Japan

**Keywords:** Gene expression, Chemokines

## Abstract

Toll-like receptor 3 (TLR3) recognizes double-stranded RNA derived from virus and its synthetic analogue, polyinosinic–polycytidylic acid [poly(I:C)]. Upon poly(I:C) binding, TLR3 activates transcription factors to express inflammatory cytokines and type I interferon. TLR3 is located in the endosomes and its recognition of poly(I:C) and activation of downstream signaling is regulated by endosomal acidification. However, the mechanism of post-transcriptional regulation in TLR3-mediated innate responses remains unclear. Here, we focused on Human antigen R (HuR, also known as ELAVL1) that recognizes and binds to the 3′ untranslated regions (3′UTRs) of target mRNAs, thereby protecting them from mRNA degradation, and found that HuR-deficient murine macrophage cells showed significantly reduced *Ifnb1* mRNA expression after poly(I:C) stimulation. HuR-deficient cells also showed a marked reduction in the expression of *Atp6v0d2* mRNA, which encodes a subunit of vacuolar-type H^+^ ATPase (V-ATPase), and therefore reduced endosomal acidification. HuR associated with the 3′UTR of *Atp6v0d2* mRNA and the stability of *Atp6v0d2* mRNA was maintained by its association with HuR. Taken together, our results suggest that HuR stabilizes *Atp6v0d2* mRNA, which is required for the TLR3-mediated innate immune responses.

## Introduction

The role of the innate immune system is to detect the presence of infectious pathogens and initiate responses to protect the host from pathogen invasion. Pathogen sensing by the host is achieved by germline-encoded pattern-recognition receptors (PRRs), which recognize conserved microbial components known as ‘pathogen-associated molecular patterns’ (PAMPs). Toll-like receptors (TLRs) are the first-investigated PRR family, which includes more than 10 functional members in mice and humans, and each member detects specific PAMPs. The recognition of PAMPs by TLRs induces the production of proinflammatory cytokines and type I interferons (IFNs). Notably, TLRs, such as TLR3, TLR7, and TLR9, are primarily localized in the endosomes, and recognizes nucleic acids such as double stranded RNA (dsRNA), single stranded RNA and DNA, respectively^1^. Infectious pathogens, such as viruses and bacteria, typically enter cells through endocytic or phagocytic pathways, and when they reach the endosomes, they are recognized by these TLRs. The endosomal localization of TLRs is essential for their activation by proteolysis and dimerization, which are controlled by several mechanisms, including the endosomal pH and transportation by UNC93b1. TLR3, TLR7, and TLR9 are synthesized in the endoplasmic reticulum (ER) and transported to the endosome with the aid of UNC93b1. Their proteolytic cleavage by cathepsins or asparagine endopeptidase is essential for the ligand-binding efficiency of the endosomal TLRs. This proteolytic activation in the endosome is thought to prevent the association of endosomal TLRs with self nucleic acids in the extracellular fluid.

TLR3 specifically recognizes dsRNA derived from viruses, as well as polyinosinic–polycytidylic acid [poly(I:C)], a synthetic agonist that mimics viral dsRNA^2,3^. TLR3 recruits TIR-domain-containing adapter-inducing interferon-β (TRIF) as its downstream adaptor protein, which subsequently activates downstream signalling pathways via the phosphorylation and nuclear translocation of the transcription factors nuclear factor κB (NF-κB) and interferon regulatory factor 3 (IRF3), leading to the expression of proinflammatory cytokines and type I IFNs, respectively^4,5^. Kinases such as IκB kinase (IKK)-related kinases, TBK1 and IKKε, are involved in activating IRF3 via the TRIF pathway. The primary outcomes of TLR3 activation are cytokine production (specifically IFN-β) and dendritic cell (DC) maturation. As well as dsRNA, herpes simplex virus (HSV) is reported to activate TLR3. The mechanism by which HSV, a DNA virus, stimulates TLR3 is still unclear. However, during infection, HSV generates an intermediate dsRNA structure, which then acts as a ligand for TLR3 activation^6,7^. Unlike the cytoplasmic PRRs namely retinoic acid-inducible gene-I-like (RIG-I-like) receptors (RLRs) including RIG-I and MDA5 that mediate recognition of viral RNA in the cytoplasm, TLR3 recognizes viral RNA via membrane bound structures, such as the endosome. It has been suggested that exogenously added dsRNA is internalized before it encounters the endosomal TLR3 in the subcellular compartment of DCs, and then activates TLR3 to elicit an antiviral response^8^.

A critical feature of mRNA, which determines its cytosolic fate, is its instability under physiological conditions, which can be manipulated through the formation of ribonucleoprotein (RNP) complexes. To form an RNP complex, RNA-binding proteins (RBPs) are required that specifically recognize either an RNA recognition motif (RRM), hnRNP K homology domain (KH), zinc fingers, or the DEAD-box helicase domain on the targeted RNA^9^. Human antigen R (HuR, also known as ‘ELAVL1’) is a ubiquitously expressed RBP. In mammals, there are four highly conserved members of this family: HuR (HuA/ELAVL1), HuB (ELAVL2), HuC (ELAVL3), and HuD (ELAVL4). HuR mainly localizes in the nucleus, but shuttles between the nucleus and cytoplasm, facilitated by its nucleocytoplasmic domains, under specific types of stimulation. The phosphorylation and methylation of HuR may play a role in initiating the HuR–mRNA interaction, triggering its translocation^10^. HuR primarily recognizes and binds to AU-rich elements (AREs). Most AREs consist of multiple copies of the specific pentameric sequence ‘AUUUA’. It has been suggested that HuR initially binds its target mRNA inside the nucleus and remains in the RNP complex to be transported into the cytoplasm, providing the mRNA ongoing protection from the degradation machinery^11^.

A previous study suggested that 44% of target mRNAs screened contained an HuR-binding site in their introns and/or 3′UTRs, suggesting that HuR interacts with mRNA before the posttranscriptional regulatory process^12^. Its co-expression at the same tissue location as another RBP protein, ARE/poly-(U) binding degradation factor 1 (AUF1), suggests that HuR competes with AUF1 for the same binding regions in specific mRNA targets^13,14^. The dynamic interaction between these two RBPs eventually determines the final cytoplasmic effect on the mRNA of interest. HuR overexpression increases the stability of ARE-containing mRNAs, possibly by inhibiting the mRNA decay pathway^15–17^. HuR, like other Hu proteins, is composed of three highly conserved RNA recognition motifs (RRMs). N-terminal RRM1 and RRM2 are essential for its recognition of and binding to the ARE in the target mRNA. The hinge region, separating RRM1 and RRM2 from RRM3 at the C-terminus, contains the HuR nucleocytoplasmic shuttling (HNS) sequence, which is crucial for the trafficking of HuR between the nucleus and the cytoplasm^18^. Although earlier studies suggested that its function is negligible, RRM3 is required for the oligomerization of HuR and promotes its interaction with its target mRNA by binding to its poly(A) tail^19^.

The innate immune response involves numerous transcriptional events, which lead to the production of various proinflammatory cytokines, IFNs, chemokines, and secretory proteins. Therefore, it requires a proper mRNA regulatory mechanism, including in the posttranscriptional phase. There have been few studies of how HuR post-transcriptionally regulates the innate immune response. Therefore, in this study, we investigated the contribution of HuR to the posttranscriptional regulation of genes involved in the innate immune system. In a previous study, we showed that HuR regulates the innate antiviral immune response by stabilizing the mRNA of polo-like kinase 2 (PLK2), which facilitates nuclear translocation of IRF3 induced by RLRs^20^. Here, we provide evidence suggesting that HuR is involved in the TLR3-mediated antiviral innate immune response by stabilizing the mRNA of *Atp6v0d2*, which encodes a subunit of vacuolar-type H^+^ adenosine triphosphatase (V-ATPase) required for the acidification of intracellular vesicles.

## Results

### Reduced poly(I:C)-induced *Ifnb* expression in HuR knockout (KO) cells

**Figure 1:**
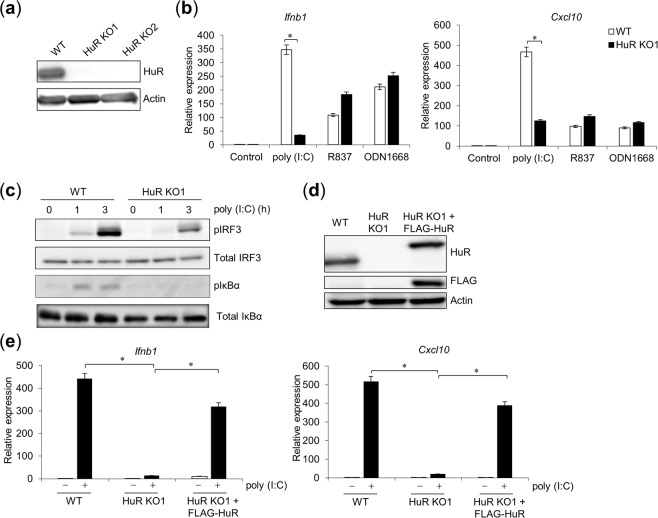
Defective response to TLR3 in HuR KO cells (**a**) Cell lysates from wild-type (WT), HuR KO1, and HuR KO2 cells were subjected to western blotting (WB) and probed with anti-HuR and anti-actin antibodies. (**b**) WT, HuR KO1, and HuR KO2 cells were stimulated with poly(I:C), R837, or ODN1668 for 8 h, and *Ifnb1* and *Cxcl10* mRNA expression were measured with RT–qPCR. (**c**) WT and HuR KO1 cells were stimulated for the indicated times, and the cell lysates were subjected to WB and probed with an anti-pIRF3, anti-IRF3, anti-pI*k*Bα or anti-I*k*Bα antibody. (**d**) HuR KO1 cells were stably transfected with FLAG–HuR-expressing plasmid with retroviral infection. Lysates from WT, HuR KO1, and HuR KO1 + FLAG–HuR cells were subjected to WB and probed with anti-FLAG, anti-HuR, or anti-actin antibody. (**e**) These cells were stimulated with poly(I:C) and the expression levels of *Ifnb1* and *Cxcl10* mRNAs were quantified with RT–qPCR. Data are the means ± SE of triplicate independent experiments. *p < 0.01, Student’s *t* test.

We previously demonstrated that an HuR KO murine macrophage cell line (RAW264.7 cells) showed reduced RLR-mediated nuclear translocation of IRF3 and reduced *Ifnb1* expression^20^. Here, we examined whether HuR KO cells have impaired responses to the nucleic-acid-sensing TLRs such as TLR3, TLR7 and TLR9. Initially, the defective expression of HuR protein in two HuR KO cell lines (KO1, KO2) was confirmed with western blotting (WB) (Fig. 1a). We then stimulated wild-type (WT) and HuR KO1 cells with poly(I:C), R837, or ODN1668, a synthetic ligand for TLR3, TLR7, or TLR9, respectively, and found that *Ifnb1* and *Cxcl10* mRNA expression was significantly reduced after poly(I:C) stimulation in the HuR KO1 cells relative to that in the WT cells. However, it was not defective in the HuR KO1 cells after R837 or ODN1668 stimulation, as measured with reverse transcription (RT)–quantitative PCR (qPCR) (Fig. 1b). Then, we performed WB to examine the phosphorylation of IRF3 and IκBα, an inhibitor of NF-κB. The phosphorylation of both IRF3 and IκBα was lower after poly(I:C) stimulation in HuR KO1 cells than in WT cells (Fig. 1c). To examine the ability of exogenous HuR to restore the response of TLR3 in KO cells, we rescued the loss of HuR by stably expressing FLAG-tagged HuR in the HuR KO1 cells. FLAG–HuR expression was confirmed with WB (Fig. 1d). Exogenous FLAG–HuR restored the expression of *Ifnb1* and *Cxcl10* mRNA after poly(I:C) stimulation to a level similar to that in the WT cells (Fig. 1e). These results suggest that HuR is required for TLR3-mediated cytokine expression in RAW264.7 cells.

### HuR knockdown reduces TLR3-mediated innate immune response

**Figure 2:**
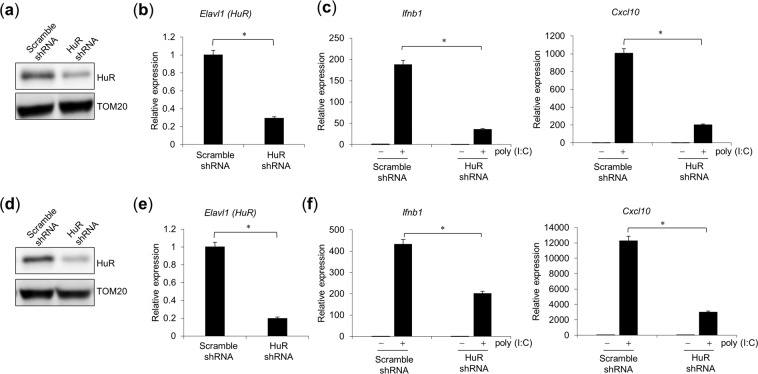
Reduced response to TLR3 in HuR knockdown cells. MEF cells (**a**–**c**) or RAW264.7 cells (**d**–**f**) were infected with a retrovirus expressing scrambled or HuR-directed shRNA and were selected with puromycin. Expression of HuR in MEF cells was confirmed with western blotting (WB) (**a**) and RT–qPCR (**b**). (**c**) MEF cells were stimulated with poly(I:C) and the expression levels of *Ifnb1* and *Cxcl10* were quantified with RT–qPCR. Expression of HuR in RAW264.7 cells was confirmed with WB (**d**) and RT–qPCR (**e**). (**f**) RAW264.7 cells were stimulated with poly(I:C) and the expression levels of *Ifnb1* and *Cxcl10* were quantified with RT–qPCR. Data are the means ± SE of triplicate independent experiments. *p < 0.01, Student’s *t* test.

To evaluate the effects of HuR protein in other cell types, we knocked down its expression in mouse embryonic fibroblasts (MEFs). MEF cells were treated with scrambled or HuR-directed small hairpin RNA (shRNA), and the reduced HuR expression was confirmed with WB using an anti-HuR antibody (Fig. 2a) and RT–qPCR (Fig. 2b). In the HuR knockdown cells, *Ifnb1* and *Cxcl10* mRNA expression after poly(I:C) stimulation was significantly reduced compared with that in the control cells (Fig. 2c). We also knocked down HuR in RAW264.7 cells (Fig. 2d,e) and found that HuR knockdown caused a significant reduction in *Ifnb1* and *Cxcl10* mRNA after poly(I:C) stimulation compared with that in cells transfected with the scrambled shRNA (Fig. 2f). These results suggest that HuR is also required for the innate immune response to poly(I:C) in MEF cells.

### *Atp6v0d2* mRNA is upregulated by poly(I:C) and regulated by HuR

**Figure 3:**
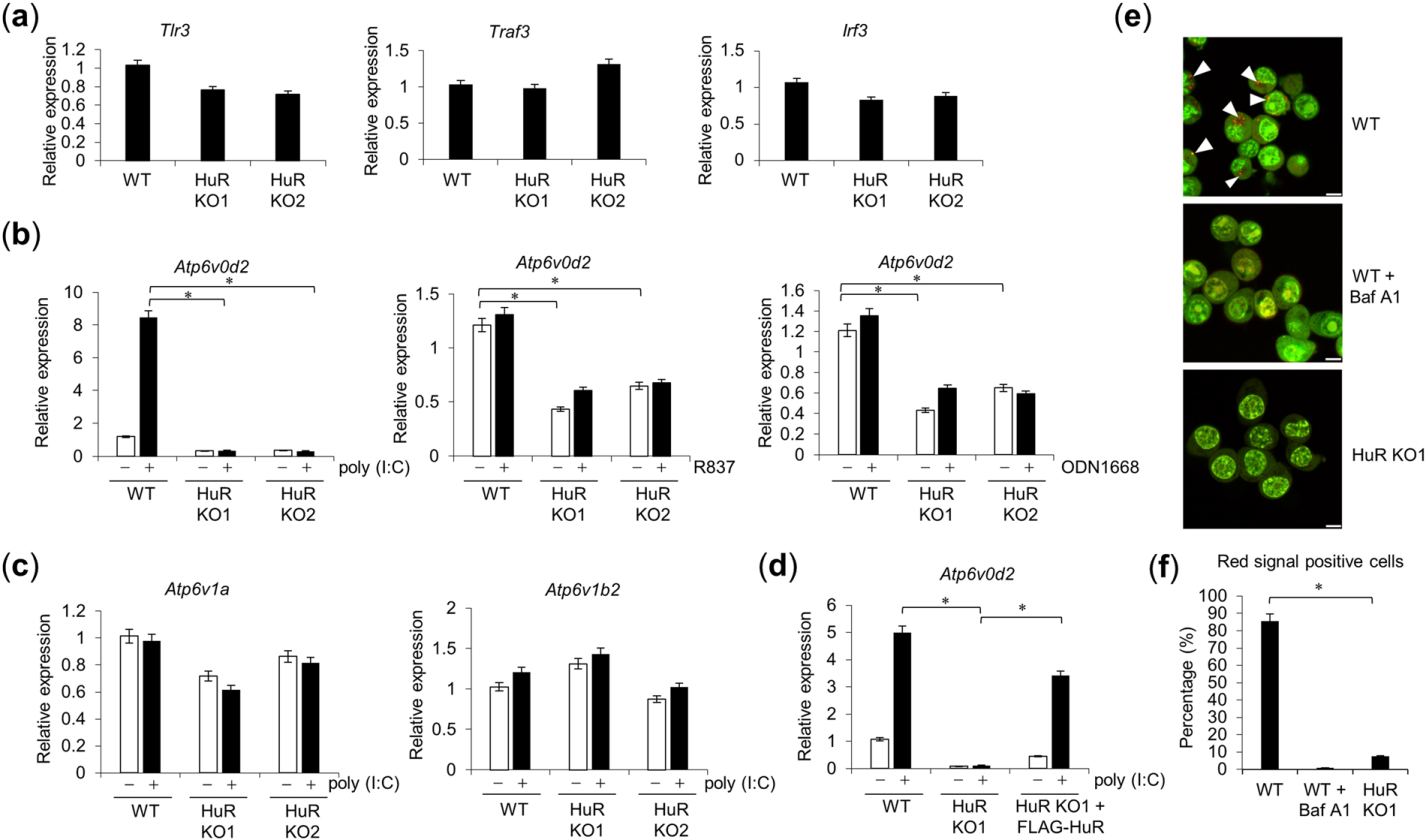
HuR regulates *Atp6v0d2* mRNA expression and endosomal acidification. (**a**) Expression for *Tlr3*, *Traf3* and *Irf3* in WT and HuR KO1 were measured by RT-qPCR (**b**,**c**) HuR KO1 and KO2 cells were stimulated with poly(I:C), R837 or ODN1668 and the levels of *Atp6v0d2* (**b**), *Atp6v1a*, and *Atp6v1b2* (**c**) were quantified with RT–qPCR. (**d**) HuR KO1 cells stably expressed FLAG–HuR after retroviral infection. Wild-type *(*WT), HuR KO1, and HuR KO1 + FLAG–HuR cells were stimulated with poly(I:C) and *Atp6v0d2* mRNA expression was measured with RT–qPCR. (**e**) Endosomal acidification was visualized with acridine orange staining. Red dots indicate acidified endosomes, highlighted with white arrowhead. Top panel: WT cells; middle panel: WT cells treated with bafilomycin A1; lower panel: HuR KO1 cells. Scale bar, 10 µm. (**f**) Number of red positive cells was counted and the percentage of positive cells was plotted as bar graph. Data are the means ± SE of triplicate independent experiments. *p < 0.01, Student’s *t* test.

HuR associates with the 3′UTR of its target mRNAs to maintain their stability^21^, so HuR deficiency is expected to reduce the expression of the target mRNAs. We measured the expression levels of TLR3 signalling molecules, including *Tlr3*, *Traf3* and *Irf3* (Fig. 3a) and *Trif*, *Traf2/6* and *Tbk1*^20^, but none of these was significantly reduced in the HuR KO cells, but we found that *Atp6v0d2*, which encodes one of the V-ATPase subunits, was reduced in HuR KO cells. We therefore measured the expression of *Atp6v0d2* mRNA in WT and HuR KO RAW264.7 cells stimulated with or without poly(I:C), R837 (TLR7 agonist) and ODN1668 (TLR9 agonist). In WT cells, the expression of *Atp6v0d2* mRNA increased after stimulation with poly(I:C). However, *Atp6v0d2* mRNA expression in the steady state was lower in the HuR KO cells than in the WT cells, and its expression after poly(I:C) stimulation was markedly reduced in the HuR KO cells, whereas it was not increased by R837 and ODN1668 stimulation (Fig. 3b). In addition, the mRNA levels of other V-ATPases subunit genes, such as *Atp6v1a* and *Atp6v1b2*, showed no significant reduction in the HuR KO cells (Fig. 3c). The expression of *Atp6v0d2* mRNA was also complemented by FLAG–HuR expression in HuR KO cells (Fig. 3d). Together, these results suggest that *Atp6v0d2* mRNA expression is maintained by HuR.

V-ATPase maintains the acidification of the endosome by proton transport. Therefore, we evaluated the effect of HuR deficiency on the acidification of the endosome using acridine orange staining, a pH indicator that stains acidic structures with 620 nm fluorescence (red) and nucleic acids with 520 nm fluorescence (green) (Fig. 3e)^22,23^. Then, the number of red signal positive cells was counted and the percentages of red signal positive cells were plotted as bar graph (Fig. 3f). WT cells showed acidic endosomal organelles in red, whereas the cells treated with bafilomycin A1, a potent V-ATPase inhibitor, showed no acidic endosomes. HuR KO1 cells showed reduced red signal (620 nm fluorescence), suggesting that V-ATPase-mediated endosomal acidification was disrupted in the HuR KO cells.

### ATP6V0D2 KO cells show a reduced TLR3 response

**Figure 4:**
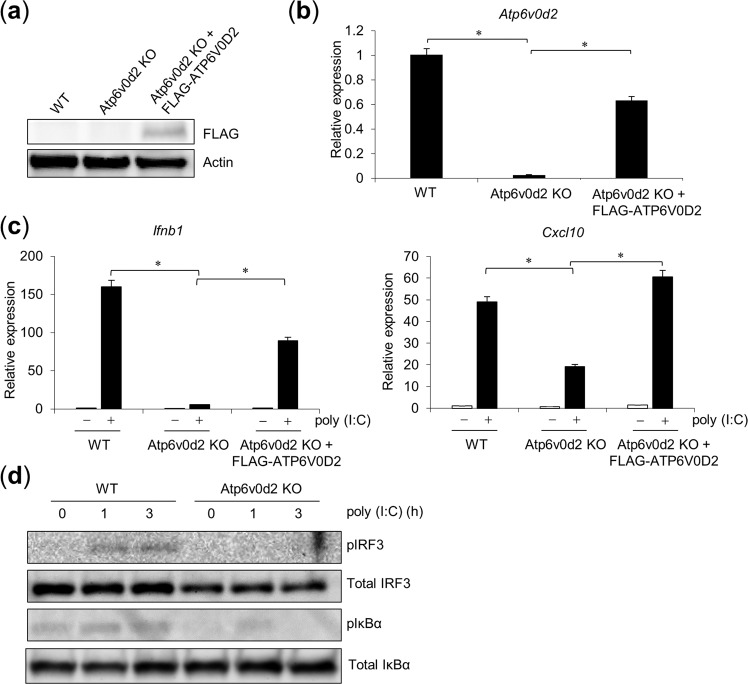
Reduced TLR3 response in ATP6V0D2 KO cells. ATP6V0D2 KO cells exogenously expressed FLAG–ATP6V0D2 after retroviral infection. After puromycin selection, *Atp6v0d2* expression was detected with western blotting (WB) (**a**) and RT–qPCR (**b**). (**c**) Following stimulation with poly(I:C) for 8 h, *Ifnb1* and *Cxcl10* expression was quantified with RT–qPCR. (**d**) WT and ATP6V0D2 KO cells were stimulated for the indicated times, and the cell lysates were subjected to WB with an anti-pIRF3, anti-IRF3, anti-pI*k*Bα or anti-I*k*Bα antibody. Data are the means ± SE of triplicate independent experiments. *p < 0.01, Student’s *t* test.

We previously established ATP6V0D2 KO RAW264.7 cells and demonstrated that these cells have reduced *Ifnb* and *Cxcl10* expression in response to ligands of TLR3, TLR7, and TLR9 than WT cells^23^. We exogenously expressed FLAG-tagged ATP6V0D2 in ATP6V0D2 KO RAW264.7 cells and measured the gene expression in response to poly(I:C) stimulation. The expression of FLAG–ATP6V0D2 was confirmed with WB and RT–qPCR (Fig. 4a,b). After poly(I:C) stimulation, the *Ifnb1* and *Cxcl10* mRNA levels were markedly reduced in the HuR KO cells, but were increased by FLAG–ATP6V0D2 expression (Fig. 4c). In addition, phosphorylation of IRF3 and IκBα after poly(I:C) stimulation was reduced in ATP6V0D2 KO cells (Fig. 4d). These results suggest that ATP6V0D2 is required for TLR3-mediated innate immune response.

### HuR associates with and stabilizes *Atp6v0d2* mRNA

**Figure 5:**
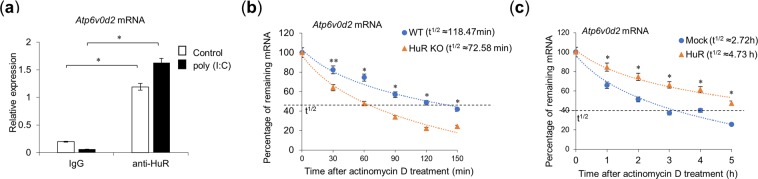
HuR associates with and stabilizes *Atp6v0d2* mRNA. (**a**) The whole-cell lysates of RAW264.7 cells treated with or without poly(I:C) stimulation for 8 h were immunoprecipitated with control-IgG- or anti-HuR-antibody-conjugated beads. The precipitated mRNA was isolated and the bead-bound *Atp6v0d2* mRNA was quantified with RT–qPCR. (**b**) Wild-type (WT) and HuR KO RAW264.7 cells were stimulated with poly(I:C) and treated with actinomycin D (2.5 µg/ml) for the indicated times. The amount of *Atp6v0d2* mRNA was quantified with RT–qPCR and normalized to the level of *Atp6v0d2* mRNA at time zero. (**c**) HEK293T cells were transiently transfected with empty or HuR-expressing plasmid and then stimulated for 8 h with poly(I:C). After the medium was changed, the cells were treated with actinomycin D (5 µg/ml) for the indicated times. The amount of *Atp6v0d2* mRNA was quantified with RT–qPCR and normalized to the amount of *Atp6v0d2* mRNA at time zero. Data are the means ± SE of triplicate independent experiments. *p < 0.01, **p < 0.05, Student’s *t* test.

To investigate whether HuR regulates the stability of *Atp6v0d2* mRNA, we used an RNA immunoprecipitation (RIP) assay. In this assay, the whole-cell lysates from poly(I:C)-stimulated RAW264.7 cells were immunoprecipitated with beads conjugated with a control or anti-HuR antibody. The *Atp6v0d2* mRNA in the precipitates was quantified with RT–qPCR. The amounts of *Atp6v0d2* mRNA in the anti-HuR antibody immunoprecipitates were significantly higher than those in control antibody immunoprecipitates (Fig. 5a). Next, we investigated the stability of *Atp6v0d2* mRNA in the presence or absence of HuR. Poly(I:C)-stimulated WT and HuR KO RAW264.7 cells were treated with a transcriptional inhibitor, actinomycin D (2.5 µg/ml), and the time-dependent changes in mRNA after the actinomycin D treatment were quantified with RT–qPCR (Fig. 5b). The half-life (t^1/2^) of *Atp6v0d2* mRNA in the WT cells was 118.47 min, whereas t^1/2^ in the HuR KO cells was 72.58 min, indicating that *Atp6v0d2* mRNA was destabilized in the HuR KO cells. We then examined whether the exogenous expression of HuR maintained the stability of *Atp6v0d2* mRNA. HEK293 cells were transfected with or without an HuR-expressing plasmid and treated with actinomycin D. t^1/2^ of *Atp6v0d2* mRNA in the HuR-overexpressing HEK293 cells was 4.73 h, whereas t^1/2^ in the mock-transfected cells was 2.72 h. Thus, within the context of a deficiency of HuR, exogenous HuR expression enhanced the stability of *Atp6v0d2* mRNA. These results suggest that HuR associates with *Atp6v0d2* mRNA and maintains its stability.

### HuR interacts with 3′UTR of *Atp6v0d2* mRNA via the HuR RRM domains

**Figure 6:**
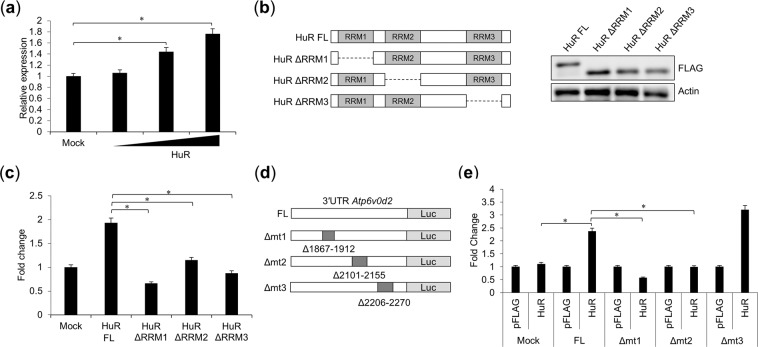
HuR interacts with 3′UTR of *Atp6v0d2* mRNA via RRMs. (**a**) HEK293T cells exogenously expressing HuR were transfected with plasmid containing the luciferase-conjugated 3′UTR of *Atp6v0d2* mRNA and the luciferase activity was measured. (**b**) Schematic structure and expression of the full-length HuR cDNA sequence or its domain deletion mutants (ΔRRM1, ΔRRM2, and ΔRRM3). (**c**) HEK293T cells were transfected with plasmid containing the luciferase-conjugated 3′UTR of *Atp6v0d2* mRNA and each ΔRRM domain mutant of HuR. (**d**) Schematic structure of the pGL3 vector containing the full-length 3′UTR of *Atp6v0d2* (FL) or its deletion mutants (Δmt1, Δmt2, or Δmt3). (**e**) HEK293T cells were transfected with plasmid containing one of the luciferase-conjugated mutants of the *Atp6v0d2* 3′UTR, with or without HuR, and the luciferase activity was measured. Data are the means ± SE of three independent experiments. *p < 0.01, Student’s *t* test.

To examine the interaction between HuR and *Atp6v0d2* mRNA, we generated a reporter plasmid in which 3′UTR of *Atp6v0d2* mRNA (nucleotides [nt] 1123–2503 of m*Atp6v0d2* mRNA) was fused with the luciferase gene. HEK293T cells were transfected with this plasmid, together with empty or HuR-expressing plasmid. HuR overexpression increased the luciferase activity in a dose-dependent manner (Fig. 6a). Because HuR contains three RRMs, we constructed deletion mutants lacking each motif and expression of each HuR deletion mutant were confirmed by WB (Fig. 6b). Then, we evaluated their effects on the recognition of the 3′UTR *Atp6v0d2* mRNA by measuring the luciferase activity and we found that these mutants failed to increase the luciferase activity, suggesting that all three individual RRMs are required to recognize the 3′UTR of *Atp6v0d2* mRNA (Fig. 6c). HuR is reported to recognize AREs in mRNAs, and the 3′UTR of *Atp6v0d2* mRNA contains three AREs. Therefore, we constructed a series of ARE deletion mutants in the 3′UTR of the *Atp6v0d2* mRNA expression plasmid, which lacked the sequence at nt 1867–1921 (Δmt1), nt 2101–2155 (Δmt2), or nt 2206–2270 (Δmt3) (Fig. 6d). HEK293T cells were transfected individually with each of these plasmids, with or without the HuR-expressing plasmid, and the luciferase activities were measured. Luciferase expression was enhanced in the cells transfected with the full-length 3′UTR of *Atp6v0d2* (FL) reporter plasmid together with the HuR-expressing plasmid, whereas it was not enhanced in the Δmt1- or Δmt2-transfected cells (Fig. 6e). The Δmt3-transfected cells showed no effect on the luciferase activity. These results suggest that the PRMs in HuR associate with the sequences at nt 1867–1921 and 2101–2155 in *Atp6v0d2* mRNA and these direct interactions are both required for the HuR-mediated stabilization of *Atp6v0d2* mRNA.

### ATP6V0D2 expression in HuR KO cells partly restores the responses to TLR3

**Figure 7:**
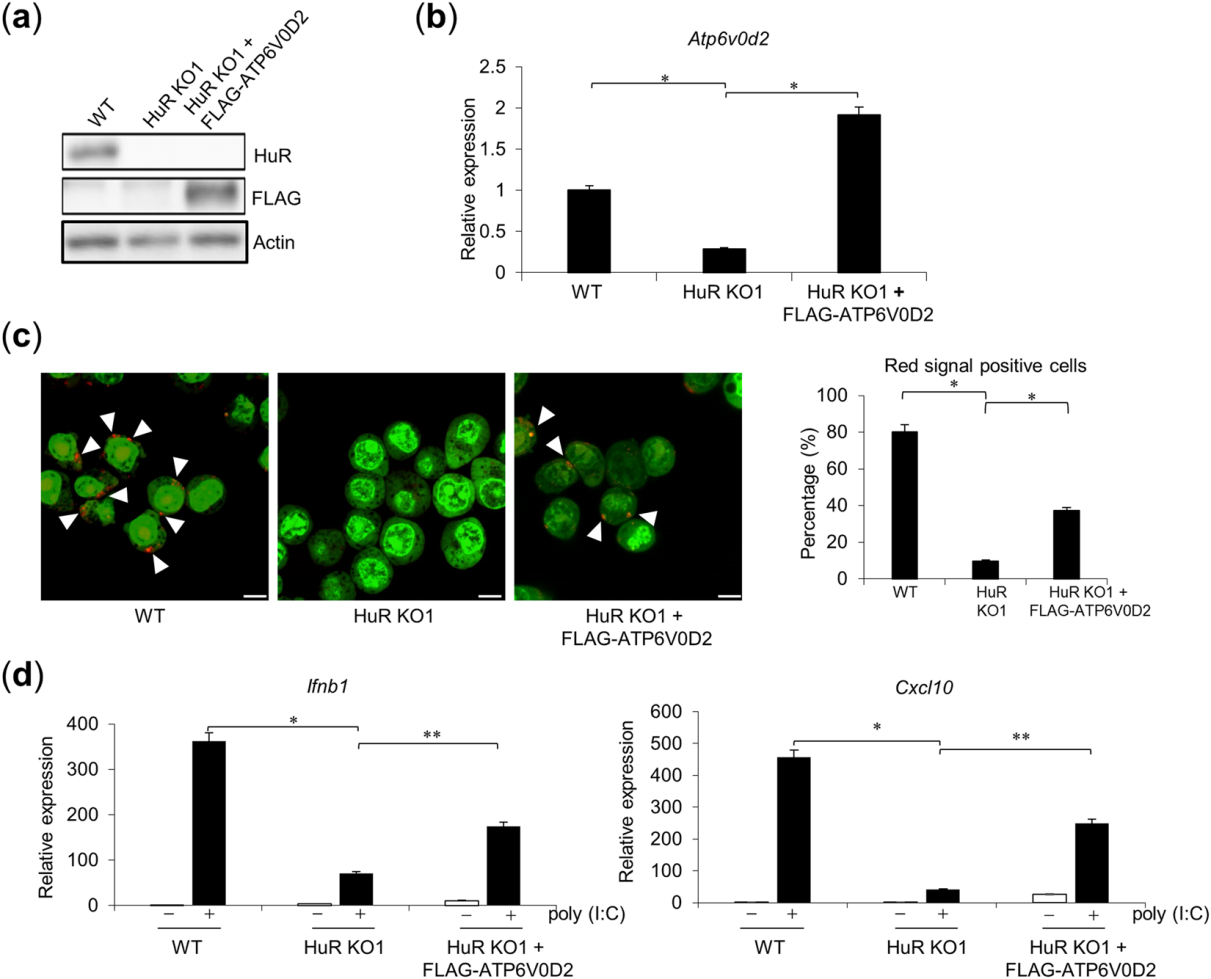
Exogenous expression of ATP6V0D2 in HuR KO cells restored their responses to TLR3. (**a**,**b**) FLAG–ATP6V0D2 was expressed in HuR KO cells after retroviral infection and its expression was confirmed with western blotting (WB) using the indicated antibodies (**a**) and with RT–qPCR (**b**). (**c**) Endosomal acidification was visualized with acridine orange staining. Red dots indicate acidified endosomes, highlighted with white arrowhead. Left panel: WT cells; middle panel: WT cells treated with Bafilomycin A1; right panel: HuR KO1 + FLAG–ATP6V0D2 cells. Scale bar, 10 µm. Number of red positive cells was counted and the percentage of positive cells was plotted as bar graph. (**d**) After poly(I:C) stimulation for 8 h, the expression levels of *Ifnb1* and *Cxcl10* mRNAs in the wild-type (WT), HuR KO1, and HuR KO1 + FLAG–ATP6V0D2 cells were measured with RT–qPCR. Data are representative of three independent experiments (means ± SE). *p < 0.01, **p < 0.05, Student’s *t* test.

We next investigated whether the reduced TLR3 responses in HuR KO cells was attributable to *Atp6v0d2* mRNA instability. To test this, we retrovirally expressed FLAG-tagged ATP6V0D2 in HuR KO RAW264.7 cells. The expression of FLAG–ATP6V0D2 was confirmed with WB using an anti-FLAG antibody and with RT–qPCR (Fig. 7a,b). In addition, we found that reduced endosomal acidification by HuR-deficiency was partially restored by exogenous Atp6v0d2 expression as determined by microscopy analysis (Fig. 7c). We then stimulated WT, HuR KO, and FLAG–ATP6V0D2-expressing HuR KO cells with poly(I:C) and measured the mRNA levels of *Ifnb1* and *Cxcl10*. *Ifnb1* and *Cxcl10* expression was significantly reduced in the HuR KO cells compared with that in the WT cells, but it was significantly upregulated in the FLAG–ATP6V0D2-expressing HuR KO cells compared with the HuR KO cells (Fig. 7c). Notably, the restoration of *Ifnb1* and *Cxcl10* expression in the FLAG–ATP6V0D2-expressing HuR KO cells was only partial compared with that in the WT cells (Fig. 7d). These results suggest that HuR at least partly regulates the TLR3-mediated innate immune responses by stabilizing the *Atp6v0d2* mRNA.

## Discussion

Several lines of evidence have suggested that the posttranscriptional regulation of mRNA plays a vital role in the gene expression involved in the immune responses. Shaw and Kamen demonstrated that the posttranscriptional degradation of granulocyte–macrophage colony-stimulating factor (*Csf2*) mRNA is mediated through its AU sequences^15^. Since then, the importance of AREs in the 3′UTR regions of the target mRNAs involved in innate immune regulation has been reported^16,17,24^. In-depth investigations of how RBPs, such as AUF1, tristetraprolin (TTP), and T-cell-restricted intracellular antigen 1 (TIA1), regulate cytokine expression and the immune responses have emphasized their importance in mRNA regulation^14,25–30^. HuR is one of the key players in this regulation, binding and stabilizing target mRNAs to allow persistent protein synthesis. It has been suggested that HuR regulates *Il4* and *Ifnb1* mRNAs^31–33^. We previously demonstrated that HuR regulates the RLR-mediated antiviral innate immune response by increasing the stability of *Plk2* mRNA, which facilitates the nuclear translocation of IRF3^20^. Here, we extended this observation by evaluating the effects of HuR deficiency on mRNA expression after stimulation with an TLR3 agonist, and found significant reductions in *Ifnb1* and *Cxcl10* mRNA expression and in IRF3 phosphorylation in poly(I:C)-stimulated HuR KO cells (Fig. 1). This was further supported by an HuR-shRNA-mediated knockdown analysis in which MEF and RAW264.7 cells showed reduced *Ifnb1* and *Cxcl10* mRNA expression after poly(I:C) stimulation (Fig. 2). Therefore, HuR regulates the antiviral innate immune response by targeting the TLR3 signalling pathway.

Activated TLR3 is known to localize in the endosomal compartment. It is transported to the endosome from the ER via the Golgi apparatus in the presence of UNC93B1 protein^34,35^. A number of studies have demonstrated that TLR3-mediated responses occur under acidic conditions, which are blocked by ATPase inhibitors, such as bafilomycin A1^36,37^. In general, ligand recognition by endosomal TLRs, such as TLR3, TLR7, and TLR9, and the subsequent activation of downstream signalling are affected by the endolysosomal pH^36,38,39^. We postulated that the downregulation of the TLR3 responses by endosomal neutralization was attributable to the impairment of TLR3 cleavage or its trafficking to the endosome. A study by Qi *et al*. (2012) demonstrated that proteolytic cleavage is not essential for TLR3 signalling in response to poly(I:C)^35^. Therefore, we reasoned that TLR3 trafficking from the ER to the endosome might be disturbed by endosomal neutralization. Because UNC93B1 is crucial for the cytosolic trafficking of TLR3, its activity in TLR3 signalling at different pHs requires further confirmation. In addition, we found that *Atp6v0d2* mRNA expression was upregulated following TLR3 stimulation whereas it was unchanged following TLR7/9 stimulation (Fig. 3b). These results suggest that poly(I:C)-mediated upregulation of *Atp6v0d2* mRNA may facilitate TLR3-mediated signaling by increasing endosomal acidification in a positive feedback fashion whereas this increment is not induced by TLR7 and TLR9. Thus, different expression profile of *Atp6v0d2* mRNA during TLR3 and TLR7/9 signaling may contribute to different responses of HuR KO cells to these TLRs.

Previously, we have shown that ATP6V0D2, a V-ATPase subunit, is required for proper cytokine expression after the stimulation of the endosomal TLRs by their ligands, including TLR3, TLR7, and TLR9^23^. A deficiency of ATP6V0D2 caused the pH of the endocytic compartment of the cell to increase, thus inhibiting the activation of the endosomal TLRs. Our RIP assay demonstrated that *Atp6v0d2* mRNA co-precipitated with an anti-HuR antibody (Fig. 5), suggesting that *Atp6v0d2* mRNA is a target for HuR binding. Because limited data are available on the subunits of ATP6V0D2 and its transcriptomic processing, we hypothesized that upon poly(I:C) treatment, *Atp6v0d2* mRNA becomes a critical target of HuR (among other transcripts) because it contains multiple AREs. These data establish that the ARE-containing *Atp6v0d2* mRNA is a candidate HuR target, whereas the exclusion of the other subunits warrants further investigation.

Our results suggest that HuR facilitates the TLR3-mediated innate immune response by stabilizing *Atp6v0d2* mRNA. The expression of FLAG–ATP6V0D2 in HuR KO cells resulted in the partial restoration of *Ifnb* and *Cxcl10* expression after poly(I:C) stimulation, supporting the notion that the defective responses of HuR KO cells to poly(I:C) are attributable to the increased degradation of *Atp6v0d2* mRNA. However, our previous study showed that HuR KO cells show reduced *Plk2* mRNA expression, which plays a role in potentiating IRF3 nuclear translocation during RLR signaling^20^. Because IRF3 is also involved in TLR3 signalling, it is plausible that TLR3-medited cytokine expression is only partially restored in FLAG–ATP6V0D2-expressing HuR KO because PLK2 expression is also reduced in these cells. Notably, HuR deficiency did not influence the induction of cytokine expression by TLR7 and TLR9 (Fig. 1b). These TLRs do not activate IRF3, but instead activate IRF7, which induces the expression of type I IFN in plasmacytoid DCs, which are known to produce vast amounts of type I IFN in response to the binding of TLR7 and TLR9. Therefore, HuR may not be essential for the activation of IRF7 during TLR7 and TLR9 signalling. However, unlike HuR KO cells, which show the normal induction of *Ifnb* and *Cxcl10* mRNAs by TLR7 and TLR9 ligands, ATP6V0D2 KO cells show defective expression of these genes^23^. Therefore, it is possible that although ATP6V0D2 expression is reduced in HuR KO cells, the acidification of the intracellular vesicles is only partially affected and insufficient to impede the robust functions of TLR7 and TLR9, such as their trafficking to endosome or proteolytic cleavage. Thus, HuR may regulate TLR3-mediated antiviral innate immunity by targeting both *Atp6v0d2* and *Plk2* mRNAs.

The posttranscriptional regulation of the TLR-mediated immune response is essential to the generation of a proper signalling cascade after stimulation by specific ligands. In the host body, particular caution is required in activating nucleic-acid-sensing TLRs, such as TLR3, TLR7, and TLR9, to avoid self-recognition and thus the induction of autoimmunity. This may be one of the reasons why all nucleic-acid sensors localize to an endocytic compartment, such as the endolysosome, to allow their activities to be monitored. A critical aspect of the regulation of nucleic-acid-recognizing TLRs is their trafficking and targeting to endosomes^38,40^. HuR, as a post-transcriptional regulator of mRNAs, seems to play some role in this regulatory mechanism. Our data suggest that HuR contributes to the endocytic regulatory machinery by targeting the ATPase subunit involved in the acidification process, which in turn governs the responsiveness of nucleic-acid-sensing TLRs, particularly TLR3. This alternative route of TLR3 activity demonstrates the complex regulatory mechanisms of TLR3, which can be manipulated to formulate new antiviral drugs for optimal effects.

## Materials and Methods

### Cells and reagents

HEK293, HEK293T, RAW264.7, and MEF cells were cultured in Dulbecco’s modified Eagle’s medium (Nakalai Tesque) supplemented with 10% heat-inactivated foetal bovine serum in a 5% CO_2_ incubator. Poly(I:C) was purchased from InvivoGen. Actinomycin D was obtained from Sigma-Aldrich. Acridine orange and bafilomycin A1 were purchased from Waldeck. Mouse anti-HuR monoclonal antibody (mAb; 3A2; Santa Cruz Biotechnology), rabbit anti-IRF3 monoclonal antibody (D83B9; Cell Signaling Technology), rabbit anti-phospho-IRF3 (Ser396) monoclonal antibody (4DaG; Cell Signaling Technology), rabbit anti-NF-κB p65 monoclonal antibody (D14E12; Cell Signaling Technology), rabbit anti-IRF3 polyclonal antibody (FL-425; Santa Cruz Biotechnology), rabbit anti-phospho-IκBα (Ser32) monoclonal antibody (14D4; Cell Signaling Technology), mouse anti-IκBα monoclonal antibody (L35A5; Cell Signaling Technology), goat anti-actin polyclonal antibody (I-19; Santa Cruz Biotechnology), and mouse anti-FLAG M2 monoclonal antibody (Sigma-Aldrich) were purchased as commercially available products.

### Plasmid construction

The full-length mouse *Elavl1* (HuR) and *Atp6v0d2* coding sequences (CDSs) were amplified with PCR from murine brain and lung cDNAs, respectively, and inserted into the pFLAG-CMV-2 expression vector from Sigma-Aldrich. The series of expression plasmids encoding HuR mutants were generated with PCR from the original full-length HuR-expressing pFLAG-CMV-2 vector. The pGL3-Promoter vector (pGL3) (Promega) containing the mouse *Atp6v0d2* 3′UTR (pGL3–*Atp6v0d2*-3′UTR) was constructed with the PCR amplification of the mouse *Atp6v0d2* 3′UTR sequence from murine thymus cDNA, which was inserted into the *Xba*l-digested pGL3 vector. Plasmids containing deletion mutants of pGL3–*Atp6v0d2*-3′UTR were generated with site-directed mutagenesis. The reporter plasmids for IFN-β and NF-κB were constructed as described previously^20,41^.

### Generation of HuR and ATP6V0D2 KO cells

HuR and ATP6V0D2 KO cells were generated as described previously^20,23^. Briefly, single guide RNA (gRNA) targeting murine *Elavl1* (HuR) exon 4 (gRNA#1: 5′-GAAGACATGTTTTCTCGGTT-3′; gRNA#2: 5′-GACCATGACACAGAAGGATG-3′) and mouse *Atp6v0d2* exon 1 (gRNA#1: 5′-GAAAATTCATCTCCAGACCA-3′) were inserted into pX330-U6-Chimeric_BB-CBh-hSpCas9 (Addgene). Partial fractions of both the murine *Elavl1*(HuR) and *Atp6v0d2* CDSs, including the gRNA-targeted site, were inserted into pCAG-EGxxFP (Addgene). RAW264.7 cells were transfected with the plasmids with electroporation, and the enhanced green fluorescent protein (EGFP)-positive cells were sorted with a FACSAria™ cell sorter (BD Biosciences) and seeded onto 96-well plates. The cells were allowed to grow for 2 weeks and the DNA was isolated. The frame-shift mutations were examined with a sequence analysis and protein expression was confirmed with WB.

### Knockdown assay

shRNAs were introduced into the *Bgl*II and *Hin*dIII sites of the retroviral vector pSUPER.retro.puro (OligoEngine), as previously described^20^. The oligonucleotide sequences used were: scrambled shRNA, 5′-CCTAAGGCTATGAAGAGATACTTCAAGAGAGTATCTCTTCATAGCCTTATTTTT-3′; and HuR shRNA, 5′-GAGAACGAATTTAATTGTCAACTTTCAAGAGAAGTTGACAATTAAATTCGTTCTC-3′. Platinum-E cells were transfected with the shRNA-carrying vectors with Lipofectamine 2000 (Life Technologies) and Opti-MEM medium (Life Technologies) in a ratio of 1:1 (μg/μl). The supernatant was filtered through a 0.22 μm filter and incubated with RAW264.7 and MEF cell. The cells were selected with puromycin (2 μg/ml) for 48 h and the surviving cells were used for the subsequent experiments.

### Western blotting (WB)

RAW246.7 and KO cells were seeded in six-well plates and stimulated with 50 μg/ml poly(I:C) for 8 h. The cells were lysed with RIPA buffer (50 mM Tris-HCl [pH 8], 150 mM NaCl, 0.1% SDS, 0.5% sodium deoxycholate, 1% Nonidet P-40). The whole-cell lysates were collected after centrifugation at 800 × g for 10 min at 4 °C, subjected to SDS-PAGE, and transferred to Immun Blot PVDF Membrane (Bio-Rad). The membrane was probed with the indicated primary antibodies, and then with horseradish-peroxidase-conjugated secondary antibody directed against mouse, rabbit, or goat IgG (Sigma-Aldrich). After incubation with Western Lightning Plus-ECL (Perkin Elmer), the membrane was exposed to ImageQuant LAS 4000 imaging system (Fujitsu Life Sciences). Image files were processed by ImageJ software 1.8.0_172 (NIH). All full-length wb are provided in Supplementary Information (Supplementary Fig. 1).

### Acridine orange staining

RAW264.7 cells were seeded in 35 mm glass-bottom culture dishes (MatTek). The cells were treated with 10 nM bafilomycin A1 for 1 h and then with freshly prepared 5 μg/ml acridine orange solution for 5 min. The cells were excited at 488 nm and emission was detected at 520 nm (green; 520 nm) as the internal control marker and at 620 nm to detect endosomal acidification (red; 620 nm) with confocal microscopy LSM 700 (Carl Zeiss). Images were collected within 1 h of the acridine orange treatment and were processed by Zen (Carl Zeiss) and ImageJ (NIH) software.

### RNA isolation and RT–qPCR

Cells were seeded in 24-well plates and stimulated with 50 μg/ml poly(I:C) for 8 h. The cells were washed with phosphate-buffered saline (PBS) and their total RNA was extracted with TRIzol Reagent (Invitrogen). To assess mRNA decay, 2.5 μg/ml actinomycin D was used to terminate transcription before RNA extraction. The total RNA was reverse transcribed to cDNA with ReverTra Ace® (Toyobo), according to the manufacturer’s protocol. Power SYBR Green PCR Master Mix (Applied Biosystems) was used for qPCR and the measurements were made with the LightCycler 96 System (Roche Diagnostics). RT-qPCR primer information was shown in Supplementary Table 1. Expressions for target genes were normalized by *Gapdh* as internal control.

### Luciferase reporter assay

HEK293T cells were seeded in 24-well plates and transiently transfected with 100 ng of reporter plasmid containing the *Atp6v0d2* 3′UTR, or one of the constructed *Atp6v0d2* 3′UTR deletion mutants, together with 500 ng of HuR expression plasmid or empty plasmid (mock) and 10 ng of pRL-TK (Promega) as the internal control. Alternatively, the cells were co-transfected with one of the HuR domain deletion mutants and the full-length *Atp6v0d2* 3′UTR, together with the internal control plasmid. The medium was replaced after 6 h. At 24 h post-transfection, the luciferase activity was measured with a TriStar^2^ LB 942 Multidetection Microplate Reader (Berthold) using the Dual-Glo Luciferase System (Promega). The targeted gene promoter activity was normalized to the *Renilla* luciferase signal.

### RNA immunoprecipitation

Antibody-conjugated protein A–Sepharose beads (GE Healthcare) were prepared by washing them in NT2 buffer (50 mM Tris-HCl [pH 7.4], 1 mM MgCl_2_, 150 mM NaCl, 0.05% Nonidet P-40). The beads were incubated with NT2 buffer supplemented with 5% bovine serum albumin at 4 °C for 2 h. After the bead slurry was washed with NT2 buffer, it was divided into two parts and incubated overnight with either the control IgG or anti-HuR antibody at 4 °C with constant agitation. RAW264.7 cells were plated in six-well plates and stimulated with 50 μg/ml LMW poly(I:C). After stimulation for 8 h, the cells were washed with PBS and suspended in polysome lysis buffer (100 mM KCl, 5 mM MgCl_2_, 0.5% Nonidet P-40, 10 mM HEPES [pH 7], 1 mM DTT, RNaseOut [Invitrogen], protease inhibitor cocktail). The cells were collected and lysed with a 26 G syringe needle (Terumo). After centrifugation at 15,300 × g for 15 min at 4 °C, the supernatants were incubated with antibody-conjugated beads for 2 h at room temperature with constant agitation. The antibody-conjugated beads were washed five times with NT2 buffer and treated with TRIzol Reagent to extract the RNA. RT–qPCR was used to measure the expression of *Atp6v0d2* mRNA.

## Supplementary information

Supplementary Table and Figure.
