# Supplementary material for: Innate immune responses through Toll-like receptor 3 require human-antigen-R-mediated Atp6v0d2 mRNA stabilization
Source: Sci Rep. 2019 Dec 31;9:20406. doi: 10.1038/s41598-019-56914-w (PMC6938500; doi:10.1038/s41598-019-56914-w)
Supplement: Supplementary file 1 — Supplementary Table and Figure. [file 41598_2019_56914_MOESM1_ESM.pdf]

Supplementary information for:

Innate immune responses through Toll-like receptor 3 require Human Antigen R-mediated ATP6V0D2 mRNA stabilization

Mohd Izwan Bin Zainol[1], Takumi Kawasaki[1]*, Warunthorn Monwan[1], Motoya Murase[1], Takuya Sueyoshi[1] and Taro Kawai[1]*

[1]Laboratory of Molecular Immunobiology, Division of Biological Science, Graduate School of Science and Technology, Nara Institute of Science and Technology (NAIST), Nara 630-0192, Japan

Running title: *Atp6v0d2* mRNA stabilization by HuR controls TLR3 signalling

*To whom correspondence should be addressed:

Takumi Kawasaki (kawast01@bs.naist.jp)

Taro Kawai (tarokawai@bs.naist.jp)

Laboratory of Molecular Immunobiology, Division of Biological Science, Graduate School of Science and Technology, Nara Institute of Science and Technology (NAIST), 8916-5 Takayama-cho, Ikoma, Nara 630-0192, Japan

Tel: +81-7-4372-5550 Fax: +81-7-4372-5539

Supplementary table 1: Primers for quantitative real-time PCR

Gene	Species	Primer sequence (5'-3')
**qPCR_mHuR_F**	Mouse	ATGAAGACCACATGGCCGAAGACT
**qPCR_mHuR_R**	Mouse	AGTTCACAAAGCCATAGCCCAAGC
**qPCR_mAtp6v0d2_F**	Mouse	TCAGATCTCTTCAAGGCTGTGCTG
**qPCR_mAtp6v0d2_R**	Mouse	GTGCCAAATGAGTTCAGAGTGATG
**qPCR_Ifnb1_F**	Mouse	ATGGTGGTCCGAGCAGAGAT
**qPCR_Ifnb1_R**	Mouse	CCACCACTCATTCTGAGGCA
**qPCR_Cxcl10_F**	Mouse	CCTGCAGGATGATGGTCAAG
**qPCR_Cxcl10_R**	Mouse	GAATTCTTGTTCGGCAGTT
**qPCR_mGAPDH_F**	Mouse	TGACGTGCCGCCTGGAGAAA
**qPCR_mTLR3_F**	Mouse	TCACTTGCTCATTCTCCCTT
**qPCR_mTLR3_R**	Mouse	GACCTCTCCATTCCTGGC
**qPCR_mTRAF3_F**	Mouse	GGTATCCTGCCCTCACAAGT
**qPCR_mTRAF3_R**	Mouse	TTCTTCTCCAGGGAGTTGCT
**qPCR_mIRF3_F**	Mouse	CTGAGGGGTTTCTGACGGAC
**qPCR_mIRF3_R**	Mouse	GCTGTGTTTTGTCCCTGTGC
**qPCR_mGAPDH_R**	Mouse	AGTGTAGCCCAAGATGCCCTTCAG
**qPCR_hATP6V0D2_F**	Human	CTTGAGTTTGAGGCCGACAG
**qPCR_hATP6V0D2_R**	Human	TGCCGAAGGTTGGATAGAGG
**qPCR_hGAPDH_F**	Human	AATCCCATCACCATCTTCCA
**qPCR_hGAPDH_R**	Human	TGGACTCCACGACGTACTCA

Supplementary figures

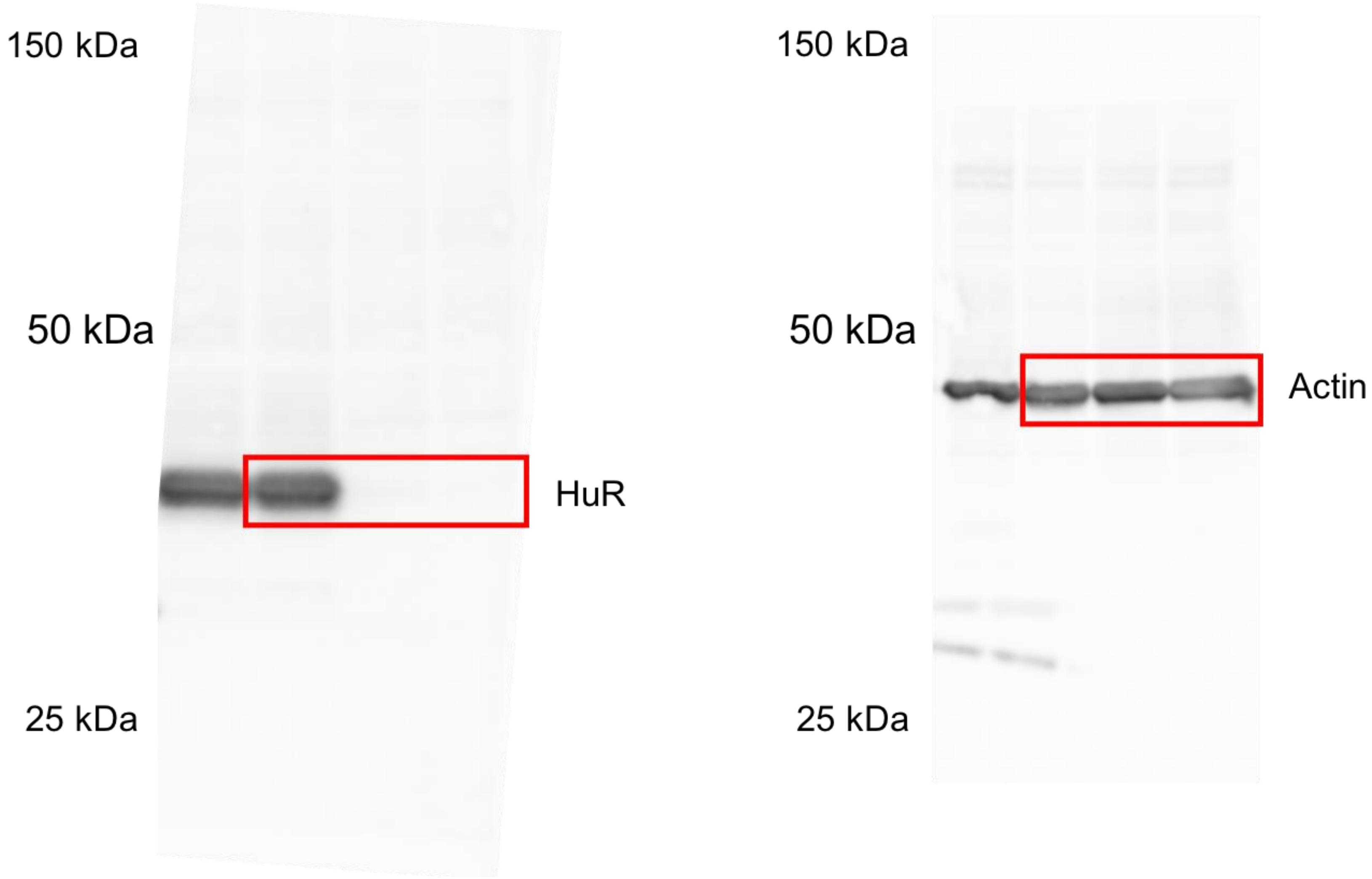

Western blot data for figure 1a

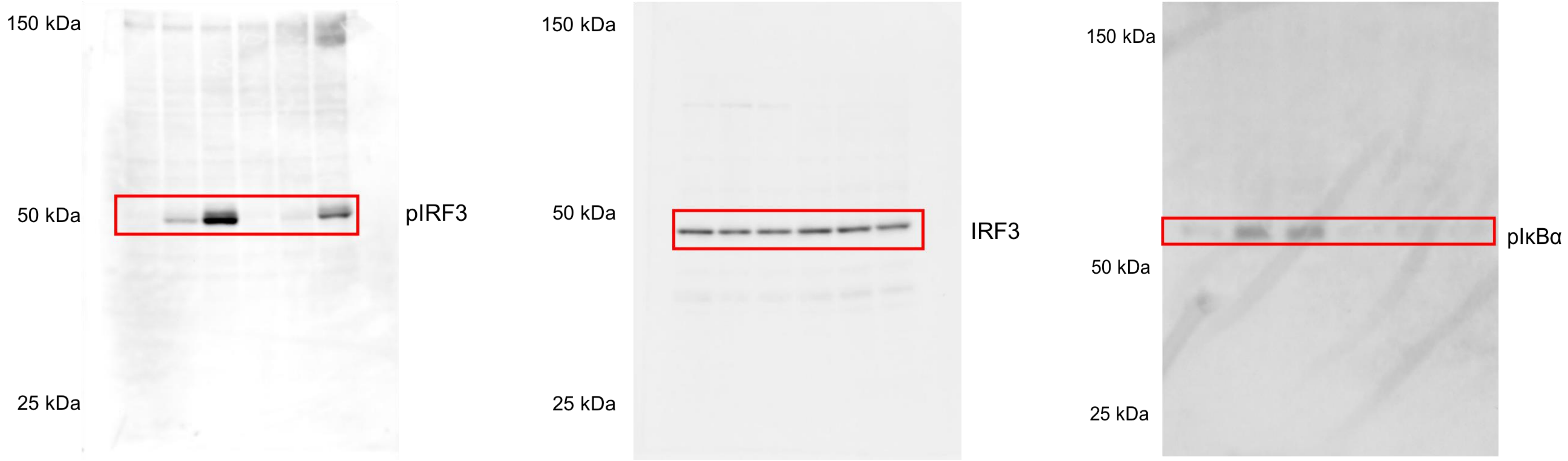

Western blot data for figure 1c

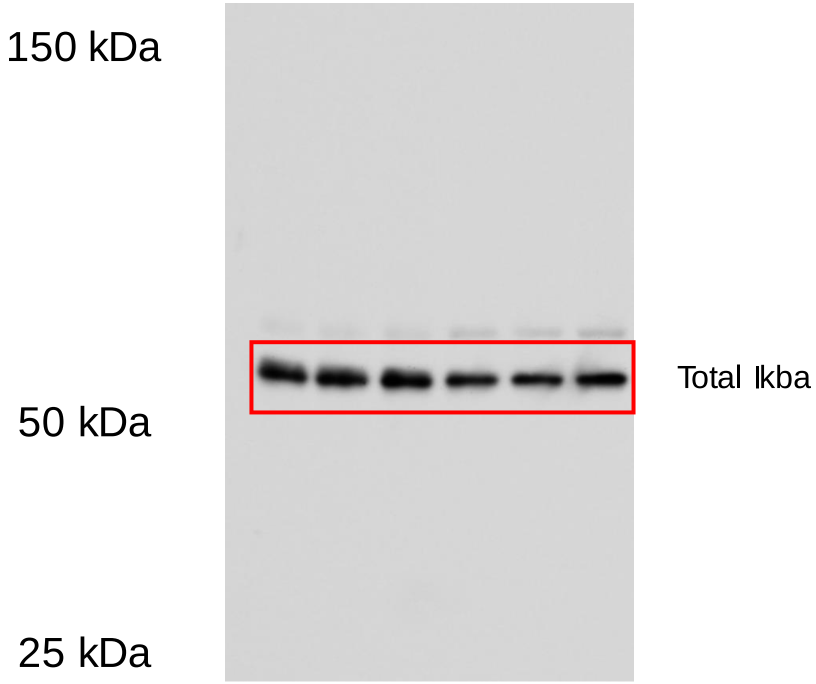

Western blot data for figure 1c

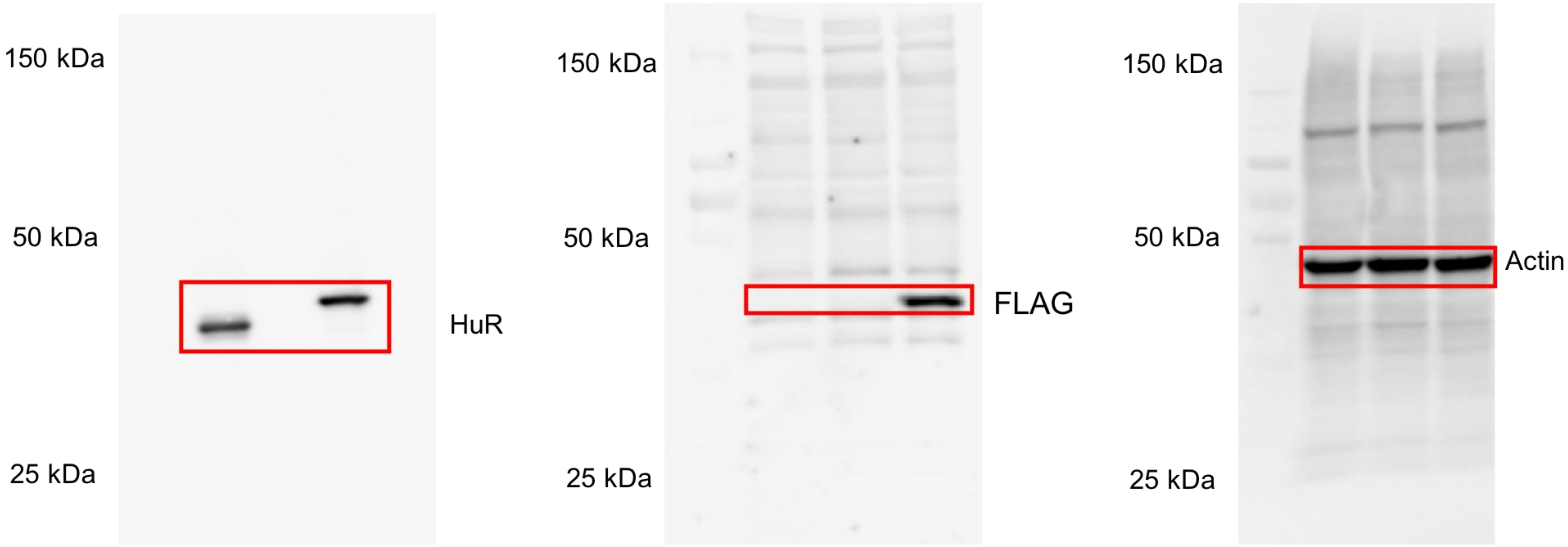

Western blot data for figure 1d

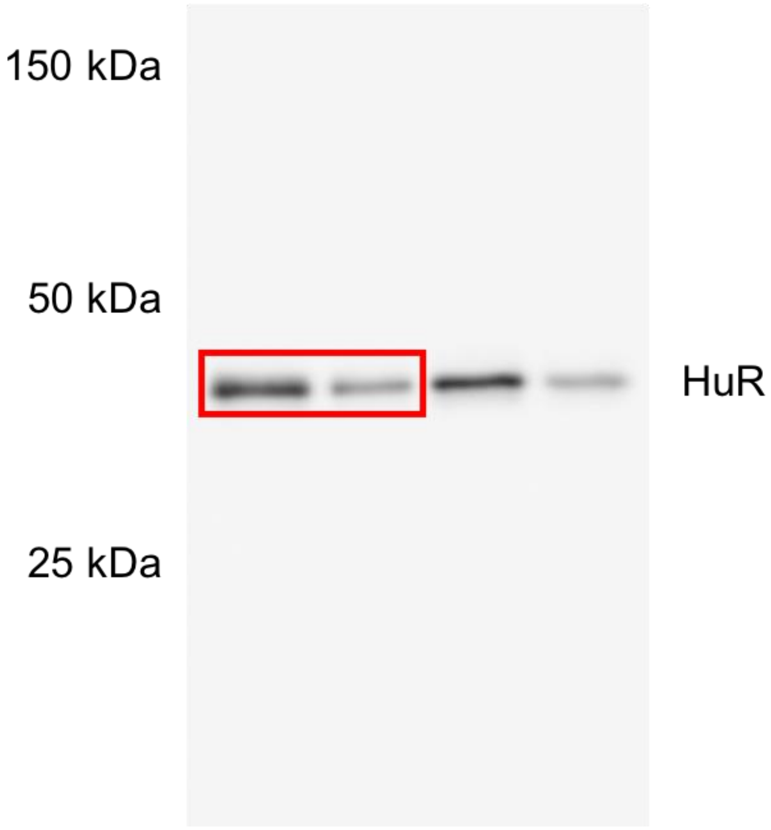

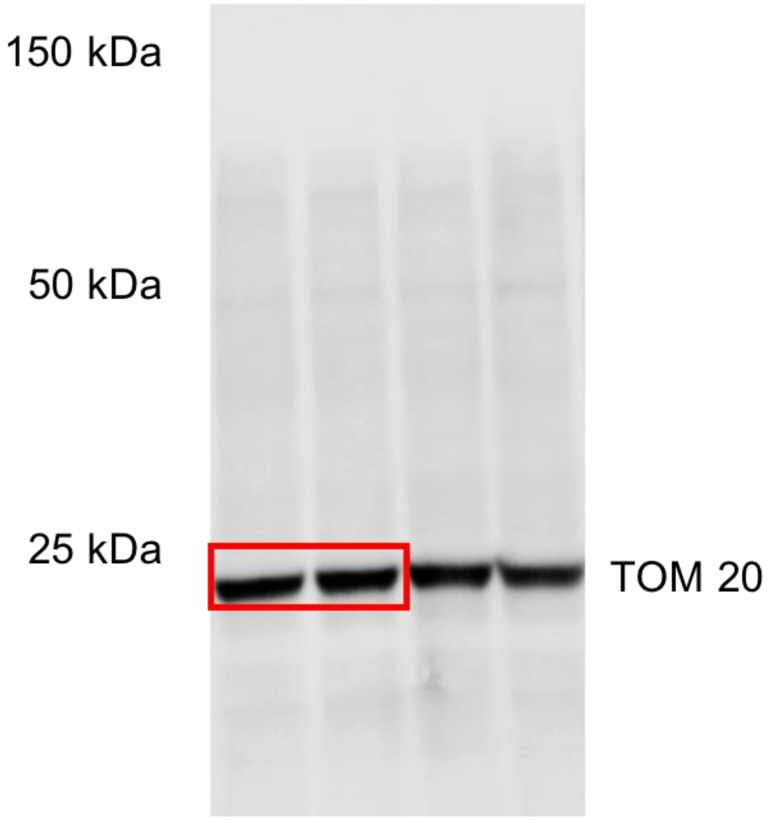

Western blot data for figure 2a

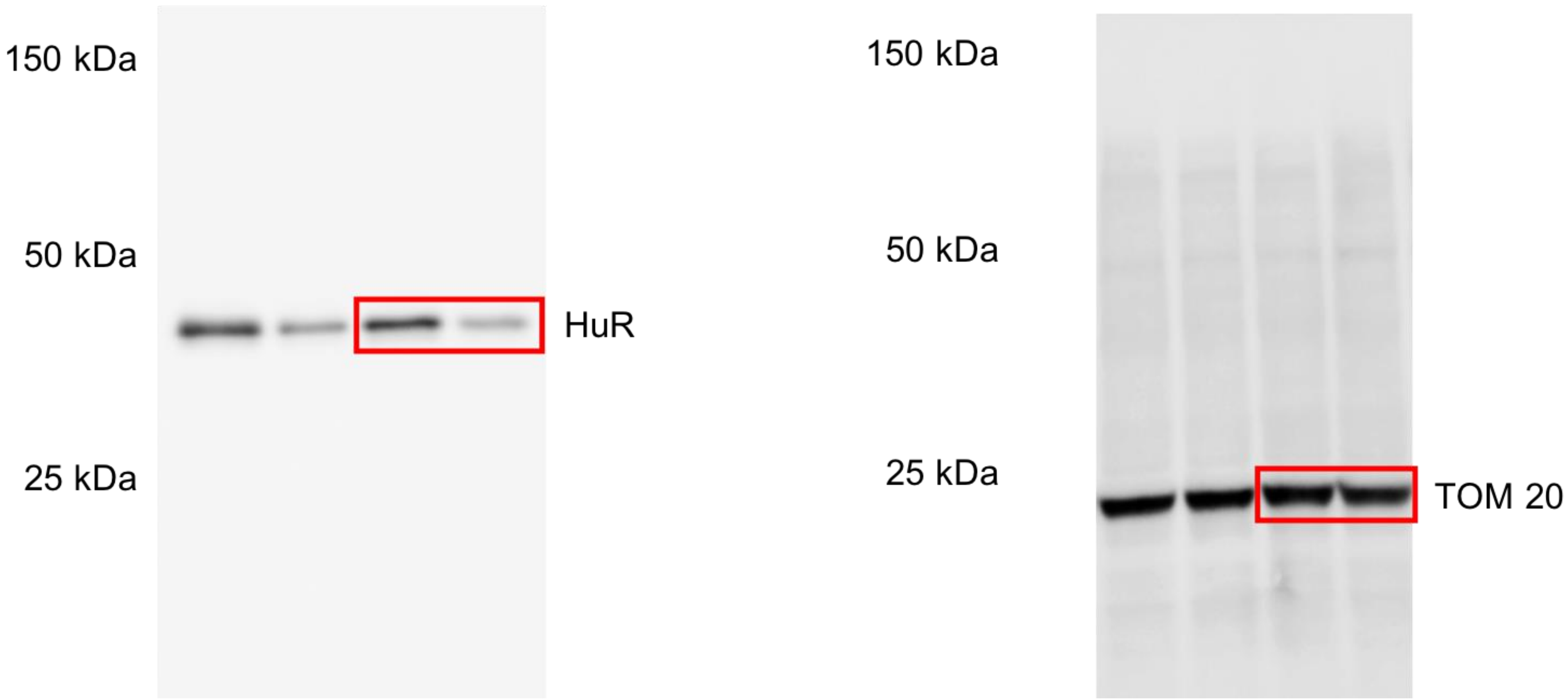

Western blot data for figure 2b

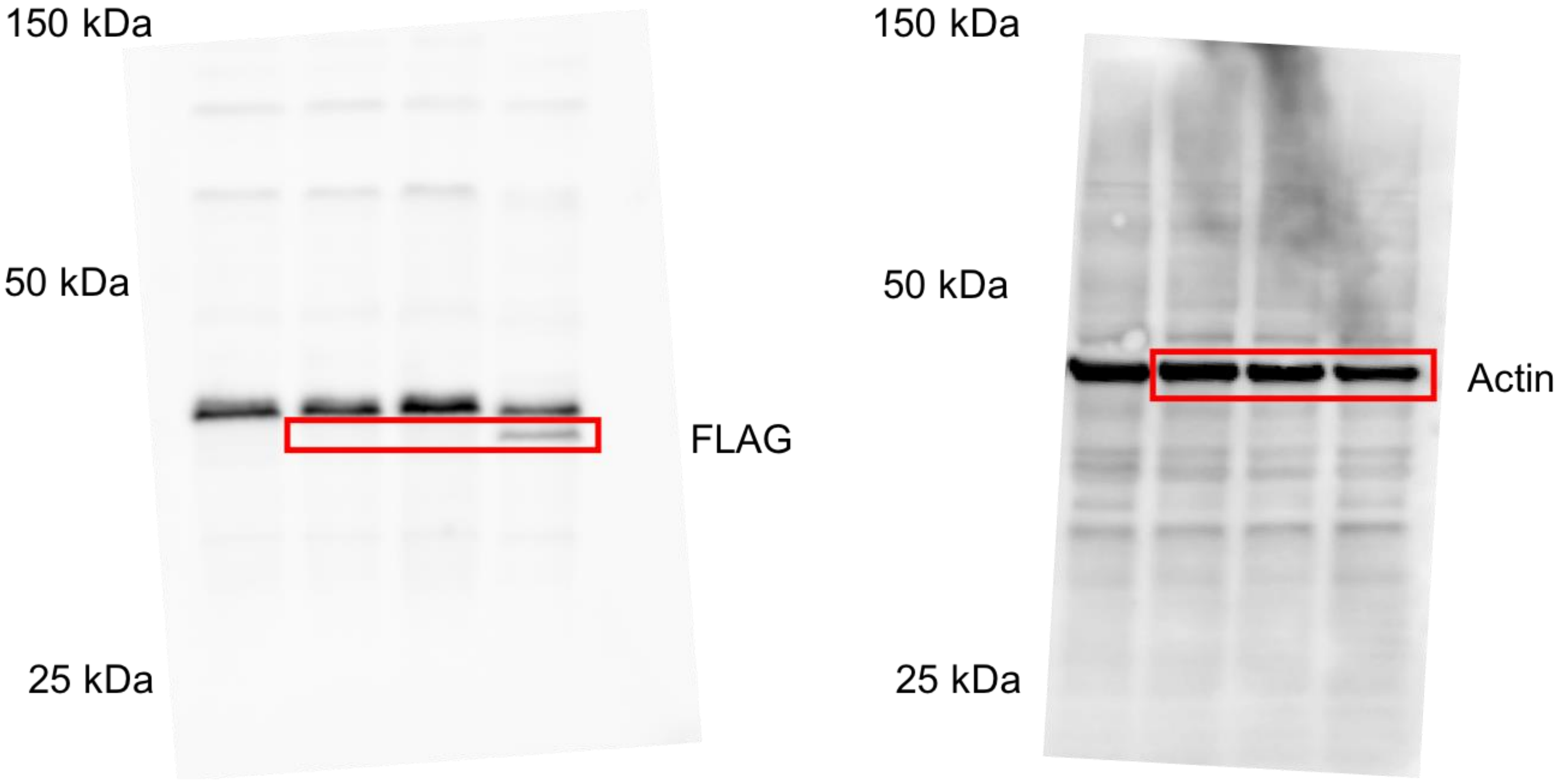

Western blot data for figure 4a

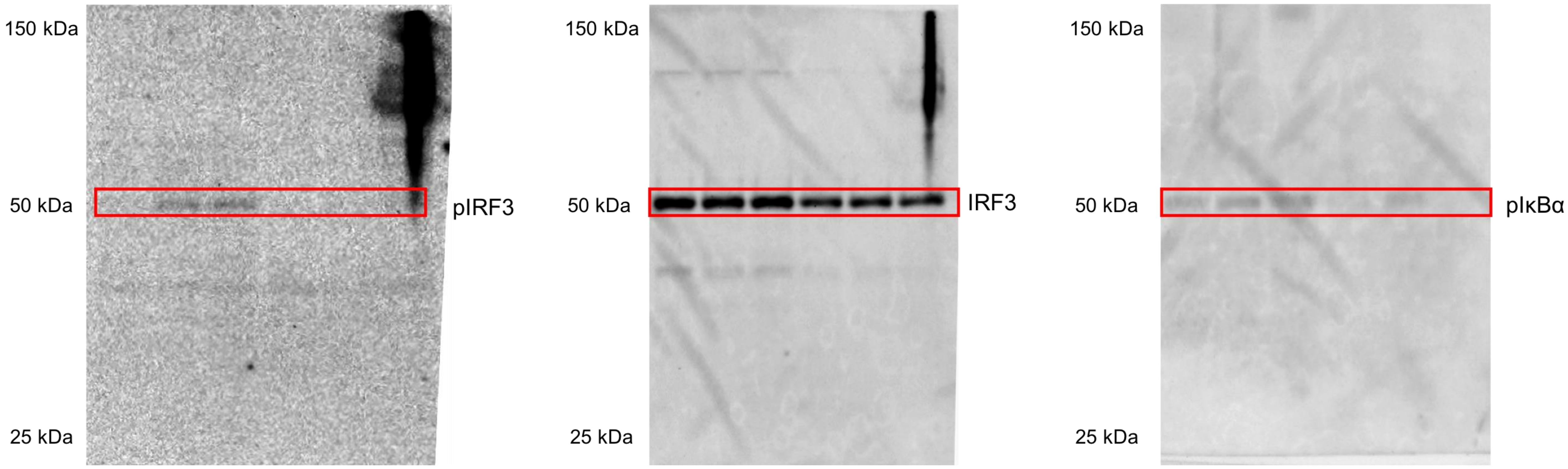

Western blot data for figure 4d

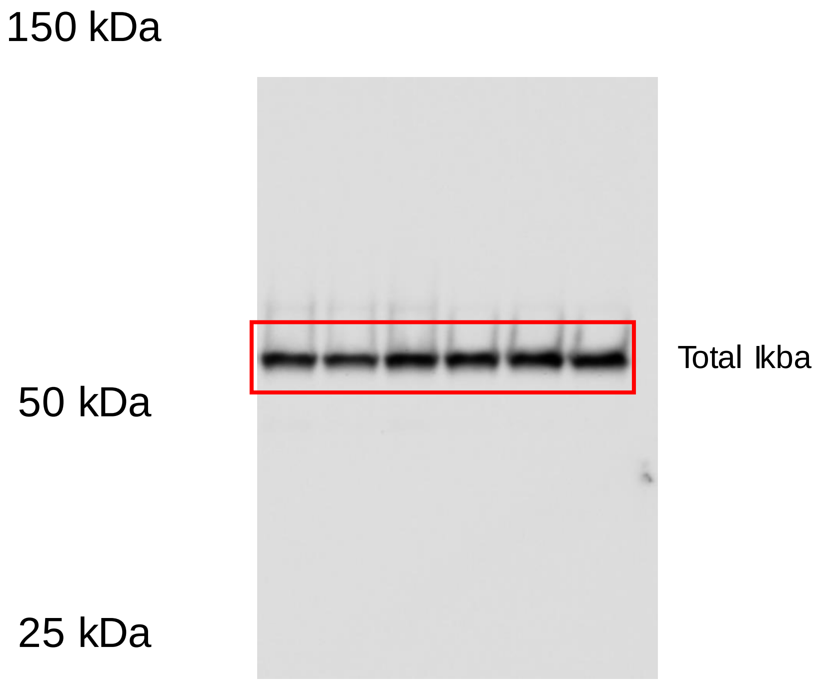

Western blot data for figure 4d

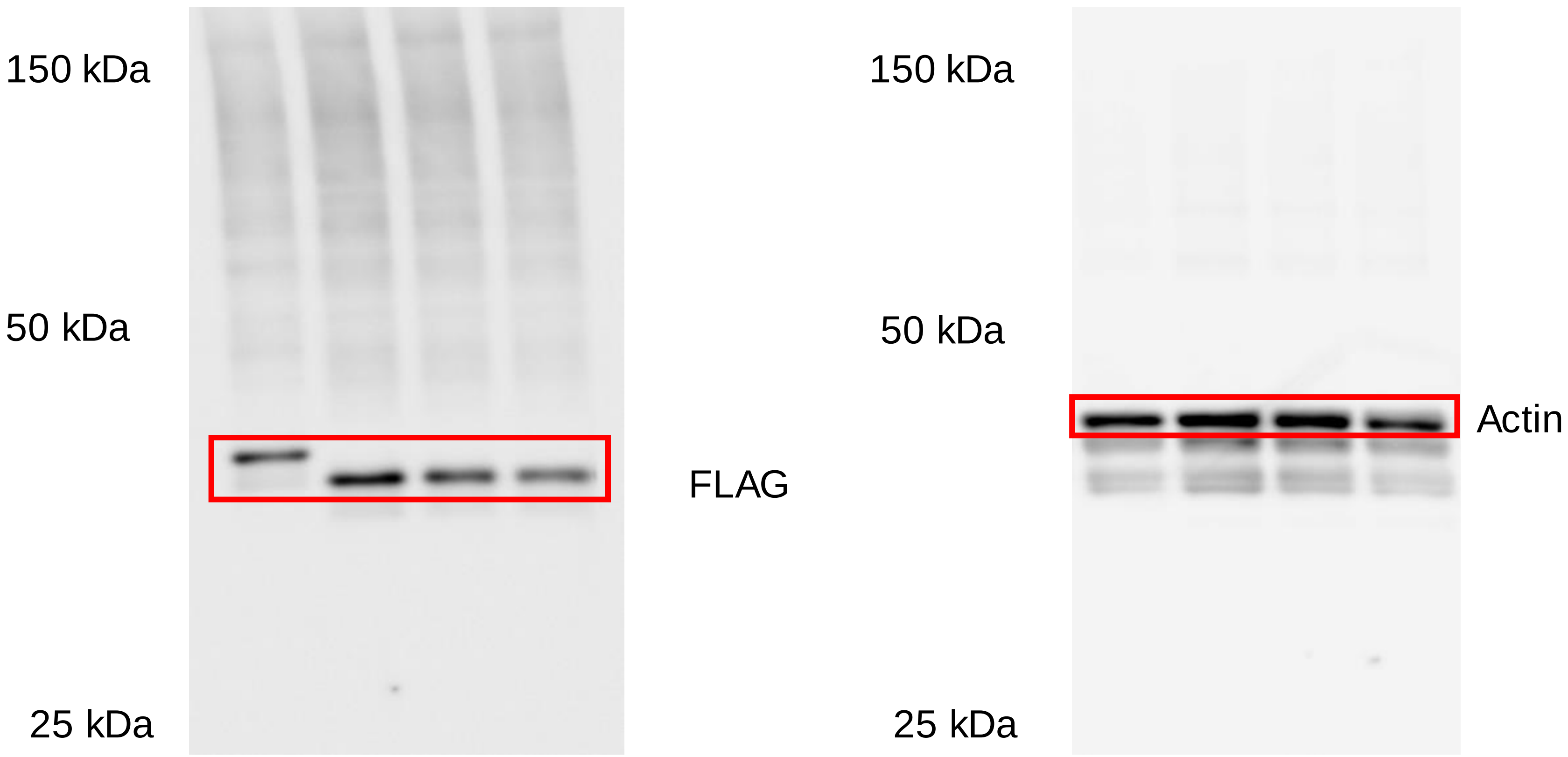

Western blot data for figure 6c

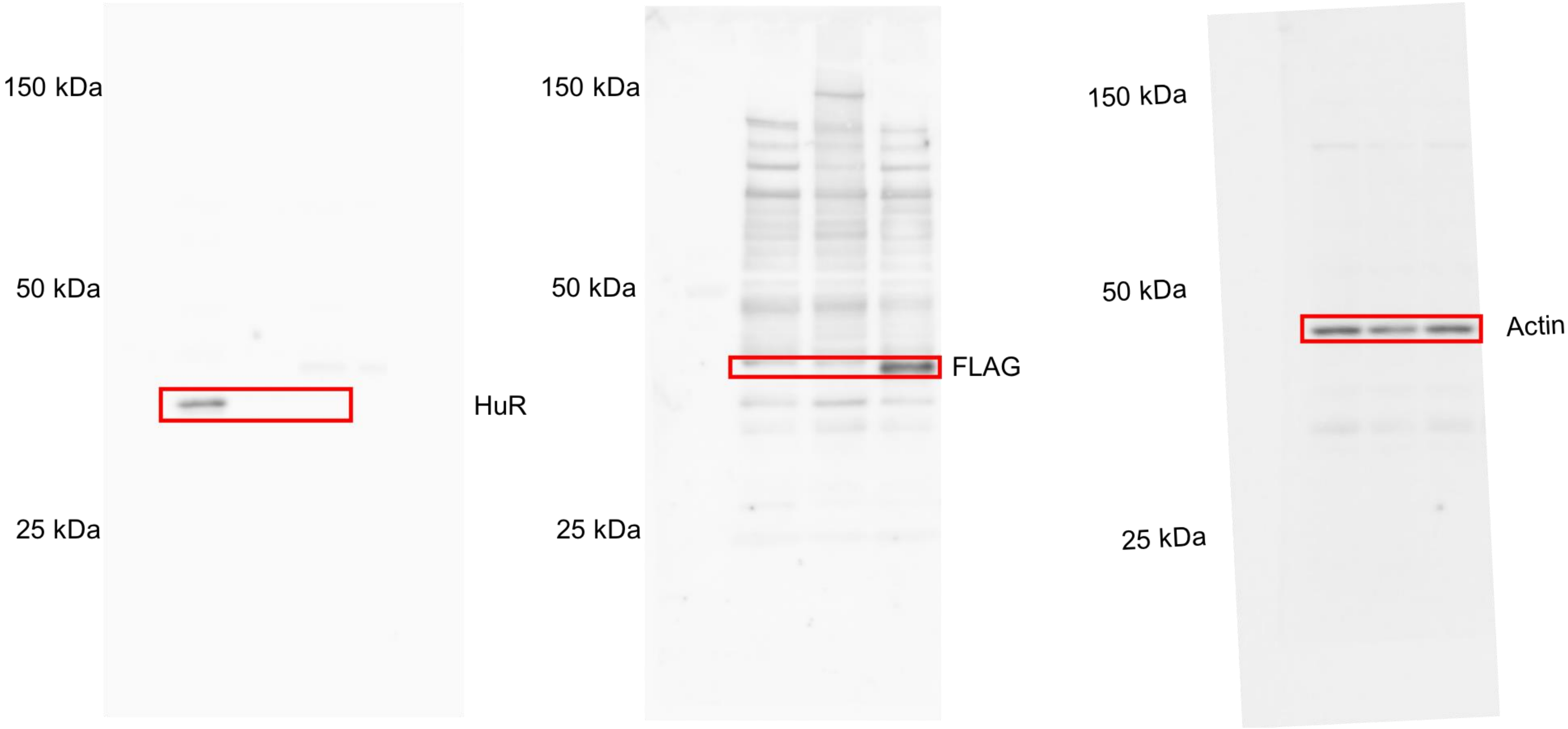

Western blot data for figure 7a
